# Targeting POLRMT by IMT1 inhibits colorectal cancer cell growth

**DOI:** 10.1038/s41419-024-07023-8

**Published:** 2024-09-03

**Authors:** Hao Wang, Yuxin Liu, Xing-sheng Lu, Yongyou Wu, Wen Gu, Guojian Yin

**Affiliations:** 1 Department of General Surgery, The Second Affiliated Hospital of Soochow University, Suzhou, China; 2 Department of General Surgery, Yancheng No.1 People’s Hospital, Yancheng, China; 3 Department of Gastroenterology, The Second Affiliated Hospital of Soochow University, Suzhou, China; 4 Departments of General Surgery, The Fourth Affiliated Hospital of Soochow University, Suzhou, China; 5 Department of General Surgery, The First Affiliated Hospital of Soochow University, Suzhou, China

**Keywords:** Targeted therapies, Colon cancer

## Abstract

This study investigates the potential anti-colorectal cancer (CRC) activity of IMT1, a novel specific inhibitor of mitochondrial RNA polymerase (POLRMT). Single-cell RNA sequencing data reveal that POLRMT is overexpressed in CRC cells. Additionally, elevated POLRMT expression was observed in local CRC tissues and cells, while its expression remained relatively low in colon epithelial tissues and cells. IMT1 significantly inhibited colony formation, cell viability, proliferation, cell cycle progression, and migration in both primary and immortalized CRC cells. Furthermore, IMT1 induced apoptosis and cell death in CRC cells. The inhibition of POLRMT by IMT1 disrupted mitochondrial functions in CRC cells, leading to mitochondrial depolarization, oxidative damage, and decreased ATP levels. Using targeted shRNA to silence POLRMT closely mirrored the effects of IMT1, showing robust anti-CRC cell activity. Crucially, the efficacy of IMT1 was diminished in CRC cells with silenced POLRMT. Contrarily, boosting POLRMT expression externally by a lentiviral construct promoted the proliferation and migration of CRC cells. Importantly, treatment with IMT1 or silencing POLRMT in primary colon cancer cells decreased the phosphorylation of Akt1-S6K1, whereas overexpression of POLRMT had the opposite effect. In nude mice, orally administering IMT1 potently restrained primary colon cancer xenograft growth. IMT1 suppressed POLRMT activity, disrupted mitochondrial function, hindered Akt-mTOR activation, and prompted apoptosis within the xenograft tissues. In addition, IMT1 administration suppressed lung metastasis of primary colon cancer cells in nude mice. These combined results highlight the robust anti-CRC activity of IMT1 by specifically targeting POLRMT.

## Introduction

Colorectal cancer (CRC), a globally pervasive malignancy [1–3], exhibits a predilection for individuals aged 50 and older, displaying considerable regional variation in its incidence [1–3]. The etiology of CRC is multifactorial, implicating lifestyle variables including dietary habits, sedentary behavior, familial history, and the presence of inflammatory bowel disease (IBD) [4, 5]. Robust evidence underscores the utility of systematic screening initiatives, designed for the early identification of CRC, in mitigating its incidence and associated mortality, particularly in select nations [1–3]. In light of its persistent prominence as a leading cause of cancer-related mortalities [1–3], the imperative for preventative measures, timely interventions, and molecularly targeted therapeutic for CRC assume paramount significance [4, 5].

Mitochondria serve as central orchestrators of cellular energy metabolism through oxidative phosphorylation (OXPHOS) in CRC [6, 7]. The malignancy of CRC is underscored by notable perturbations in mitochondrial homeostasis, encompassing variations in mitochondrial DNA (mtDNA) content, the emergence of mtDNA mutations, and disruptions in the expression of pivotal mitochondrial genes [8–10]. These aberrations exert substantial influence on the progression of CRC, impacting critical cellular processes, including energy metabolism, cell proliferation, and apoptotic mechanisms [8–12].

RNA polymerase mitochondrial (POLRMT) serves as a crucial enzyme responsible for transcribing mtDNA in eukaryotic cells [13–16]. POLRMT is located in mitochondria and assumes a pivotal role in generating essential mitochondrial RNA molecules, which are subsequently employed in the production of vital components for the respiratory chain [13–16]. These components are integral to the energy-producing functions of the mitochondria, rendering POLRMT indispensable for cellular energy metabolism [13–16]. Any disruption or malfunction of POLRMT has been associated with a range of mitochondrial disorders and diseases, such as cancer [17–20].

Recent studies have proposed a pivotal role of POLRMT in cancer growth and progression. Zhou et al., demonstrated an increased expression of POLRMT in human non-small cell lung cancer (NSCLC) tissues and cells, highlighting its importance in cancer cell growth [17]. The use of POLRMT shRNA or CRISPR/Cas9 knockout (KO) effectively impeded mtDNA transcription and inhibited the growth of NSCLC cells in vitro and NSCLC xenografts in nude mice [17]. Han and colleagues unveiled the overexpression of POLRMT in osteosarcoma (OS), causing enhanced mitochondrial functions and the promotion of OS cell growth [18]. Notably, genetic silencing of POLRMT, achieved through shRNA or CRISPR/Cas9 methodologies, exhibited a potent inhibitory effect on the growth of OS cells in vitro and in vivo [6].

The above results implied that directing interventions towards POLRMT might lead to a promising anti-cancer effect. IMT1 is a first-in-class and potent allosteric inhibitor of POLRMT, demonstrating remarkable specificity [19, 21]. It effectively hinders the transcription and expression of mtDNA, resulting in the disruption of the OXPHOS system and reduced production of mitochondrial energy (ATP) [19, 21]. Notably, IMT1 exhibits significant efficacy in preclinical mouse models, showing its potential as a promising treatment for cancer [22, 23]. However, investigations into the activity of IMT1 in CRC and the associated underlying mechanisms remain unexplored.

## Materials and methods

### Chemicals and reagents

Polybrene, *N*-acetylcysteine (NAC), ATP, and puromycin were sourced from Sigma-Aldrich (St. Louis, MO). IMT1 was acquired from Dr. Wang [22, 23]. The pan-caspase inhibitor (zVAD-fmk) and the specific caspase-9 inhibitor zDEVD-fmk were procured from Calbiochem (Darmstadt, Germany). Antibodies were purchased from Cell Signaling Technology (Beverly, MA) and Santa Cruz Biotech (Santa Cruz, CA). Fluorescence probes such as TUNEL, JC-1, DAPI, MitoSOX, EdU, and propidium iodide (PI) were obtained from Thermo-Fisher Invitrogen (Shanghai, China).

### Cells

Primary human colon cancer cells (pCan1/2/3) sourced from three primary patients with written informed consent, along with primary human colon epithelial cells (pEpi1/2) obtained from two healthy donors, were previously detailed in our prior publication [24]. These cells were generously provided by Dr. Lu from Nanjing Medical University [25–27]. Cultivation of the primary human cells in the specified medium was outlined in early studies [25, 28]. Additionally, the established HCT116 CRC cells were also provided by Dr. Lu [25–27]. Regular examination for mycoplasma and microbial contamination was conducted every two months across all established and primary cells. Genotypic validation was ensured through routine STR (short tandem repeat) profiling, assessment of population doubling time, and monitoring of cell morphology. The study protocols adhered to the ethical guidelines outlined by the Ethics Committee of Soochow University, in compliance with the Declaration of Helsinki.

### Human tissues

Human colon cancer tissues, along with matched adjacent normal colon epithelial tissues, were from a total of twenty primary colon cancer patients, all of whom underwent tumor resection surgeries at our institutions and provided written informed consent. These fresh tissues were preserved in liquid nitrogen. Tissue lysates underwent tests through quantitative real-time PCR (qRT-PCR) and Western blotting assays. The protocols were approved by the Ethics Committee of Soochow University and strictly adhered to the principles outlined in the Declaration of Helsinki.

### Immunohistochemistry (IHC)

The paraffin-embedded xenograft sections underwent a series of treatments including baking, dewaxing, and hydration. Afterward, these sections were cleansed using a 0.4% Triton X-100 in PBS (PBST) solution, followed by a 20-min incubation in 10% serum in PBST to minimize nonspecific binding. Subsequent steps involved blocking endogenous peroxidase activity with hydrogen peroxide and applying a tissue Ki-67 staining kit (Biyuntian, Wuxi, China). Finally, visualization was achieved using diaminobenzidine (DAB) staining.

### Genetic modification of POLRMT

POLRMT shRNA or overexpression experiments utilized lentivirus provided by Dr. Shi from Soochow University [17]. CRC cells were seeded at 60% confluence in a complete medium containing polybrene and then infected with lentivirus at an MOI (multiplicity of infection) of 10–11. After 48 h, cells were cultured in a fresh medium supplemented with puromycin (5 μg/mL). Stable cells expressing either POLRMT shRNA or the PLORMT overexpression construct were established within another 5–6 passages. Control cells underwent infection with lentivirus containing either scramble control shRNA (“shC”) or an empty vector (“Vec”). Continuous monitoring of PLORMT expression in these cells was conducted through qRT-PCR and Western blotting assays.

### Colony formation

For colony formation, cells were initially seeded at 30,000 cells per well in a 10-cm culturing dish and maintained in complete medium with serum (8%), with renewal every 2 days. After 14 days, cell colonies were fixed, stained, and manually counted.

### Cell migration assays

In vitro cell migration was assessed using “Transwell” chambers (12-µm pore size, Corning). Briefly, 12,000 cells per chamber, re-suspended in serum-free basic medium, were coated on the upper chamber surface. The lower compartment received 600 µL of serum (8%)-containing complete medium. After 16 h, cells on the upper surfaces were gently scraped, and those that migrated to the lower chamber membranes were fixed and stained.

### Caspase activity

Caspase-3/-9 activities were assessed via commercial kits (Abcam, Shanghai, China). Cells were seeded into 96-well plates at 3500 cells per well and subjected to the designated treatments. Afterward, cell lysates were incubated with the loading solution containing the caspase-3/-9 substrate for 50 min. Subsequently, caspase-3/-9 activities were determined using a fluorescence microplate reader (BioTek Synergy) at 625 nm emission for caspase-3 and 455 nm emission for caspase-9.

### Fluorescence dye assays

Cells subjected to the specified treatments were seeded into twelve-well plates and were cultivated for the designated duration. Following this, cells underwent fixation, permeabilization, and washing, and were subsequently exposed to the specified fluorescence dyes. After PBS wash, the fluorescence signals were captured using a Zeiss confocal microscope, and their intensities were quantified.

### Glutathione (GSH) to oxidized glutathione (GSSG) ratio

The reduced (GSH) to oxidized (GSSG) ratio serves as an indicator of cellular redox status, with a higher ratio reflecting a reducing environment, and a lower ratio indicating increased oxidative stress. A GSH/GSSG kit from Biyuntian (Wuxi, China) was used. Cellular or tissue lysates were mixed with DTNB, glutathione reductase, and NADPH (attached in the kit). Subsequently, these lysates were mixed with a reaction solution, and the spectrophotometer measured absorbance at 445 nm for 5 min. The concentrations of GSH and GSSG in the lysates were determined by creating a standard curve using GSH and GSSG standards, and the ratio was normalized based on the protein concentration.

### Single-strand DNA (ssDNA) ELISA

The cell lysates were assessed using an ssDNA ELISA kit from Invitrogen (Shanghai, China). The optical density (OD), indicating ssDNA intensity, was measured at 450 nm in individual wells during the ELISA analysis.

### ATP contents and the mitochondrial complex I activity

The lysates from the treated cells or the described xenograft tissues underwent analysis using an ATP assay kit (Biyuntian, Wuxi, China) and a mitochondrial complex I activity kit (Biyuntian, Wuxi, China). Subsequently, the cellular ATP levels and mitochondrial complex I activity were assessed following the protocols provided with each kit.

### Thiobarbituric acid reactive substance (TBAR) assay

The intensity of lipid peroxidation (testing 30 μg of cellular/tissue lysates per treatment) was evaluated using a TBAR kit from Cayman Chemical (MI), following the provided protocols. The TBAR reaction reagent enabled the colorimetric quantification of lipid peroxidation, measuring the intensity of malondialdehyde. The TBAR intensity was assessed at 545 nm of reference wavelength, and the resulting value, in nmol per mg of total protein, was normalized against the control.

### Akt1 mutation

The initial step involved seeding cells onto six-well plates at 50–60% confluence in a complete medium containing polybrene. Subsequently, these cells were transduced either with a lentiviral construct containing the S473D constitutively active mutant of Akt1 (caAkt1, obtained from Dr. Xu [29], lacking a tag) or with an empty vector. Post-selection using puromycin, stable cell populations were established, and the presence of caAkt1 expression was confirmed within these stable cells.

### Other assays

Additional assays, such as Western blotting, qRT-PCR, CCK-8 cell viability assessment, and Trypan blue staining for cell death intensity, as well as apoptosis assays, have been extensively documented in our prior studies [12, 24, 30–32]. The cytochrome C ELISA assays have been described in detail elsewhere [33, 34]. Figure S1 lists uncropped blotting images of the study.

### Xenograft assays

The protocols were reported in our previous studies with minor changes [24, 30]. Briefly, the primary colon cancer cells (pCan1) were injected subcutaneously into nude mice (half male half female, 18.3–18.5 g, 4–5-week-old) at a concentration of seven million cells per mouse in 0.3 mL FBS-free DMEM/Matrigel medium. Subcutaneous pCan1 xenografts developed within 21 days, each reaching a volume close to 100 mm [3]. The mice bearing xenografts were subsequently orally administered with either IMT1 or a vehicle control [22, 35]. IMT1 was given at a dose of 50 mg/kg body weight for two cycles (on Day 0 and Day 3). The xenograft volumes were measured every six days using the described methods [36]. The protocols of TUNEL-fluorescence staining xenograft sections were reported previously [35]. The lung metastasis model was also established. Briefly, pCan-1 cells were harvested, washed, and resuspended in a serum-free medium. A total of 2 × 10^6^ cells in 100 μL of the suspension were injected into the lateral tail vein of 6-week-old nude mice. The mice were monitored daily for signs of distress and were sacrificed at 40 days post-injection to assess metastatic burden. Lungs were excised, rinsed in PBS, and fixed in 10% formalin. Lung tissues were embedded in paraffin, sectioned, and stained with hematoxylin and eosin to visualize and quantify metastatic lesions. The protocols involving animals received approval from both the Institutional Animal Care and Use Committee and the Ethics Committee of Soochow University.

### Statistical analysis

In vitro experiments were replicated five times, and the data exhibited a normal distribution, presented as mean ± standard deviation (SD). Statistical analyses were performed using SPSS 23.0 software (SPSS Co., Chicago, IL). The unpaired Student’s *t*-test was employed for comparing two specific groups, while one-way ANOVA with the Scheffe’ and Tukey Test was used for comparisons involving more than two groups. *P* values less than 0.05 were considered statistically significant and were denoted by an asterisk (*) or pound sign (^#^).

## Results

### The single-cell RNA sequencing (scRNA-seq) data reveals POLRMT overexpression in CRC cells and cancer-associated endothelial cells

**Fig. 1:**
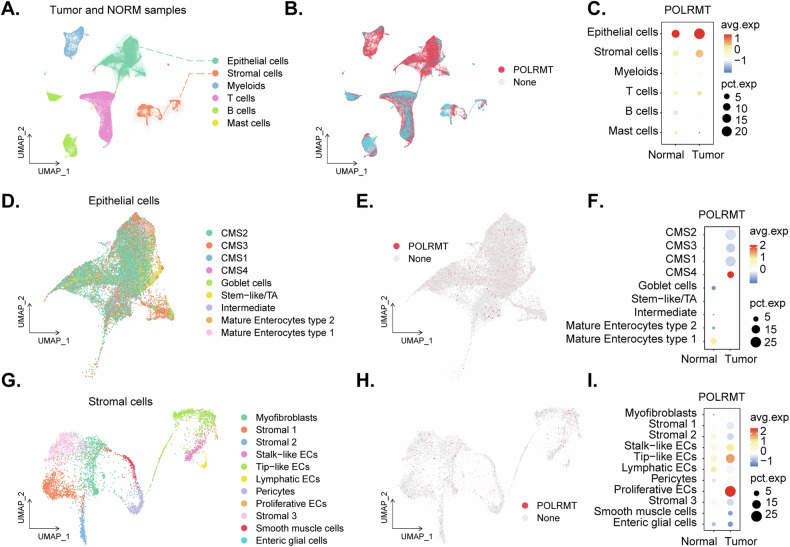
The scRNA-seq reveals POLRMT overexpression in CRC cells and cancer-associated endothelial cells. Single-cell data analysis of CRC from the GSE132465 dataset, with cell annotations provided by the original authors (**A**, **B**). Dot plot illustrating the distribution of *POLRMT* in different cells of CRC and normal tissues, showing increased expression in the cancer group (**C**). The epithelial cell subgroup of CRC and normal tissues was extracted (**D**) and expression of *POLRMT* in different subgroups was shown (**E**, **F**). The stromal cell subgroup was also extracted from CRC and normal tissues (**G**) and expression of *POLRMT* in different subgroups was shown (**H**, **I**).

We first analyzed available scRNA-seq data from CRC [37], sourced from the publicly available dataset GSE132465. Cell annotations provided by the original authors [37] were utilized for the identification of distinct cell populations (Fig. 1A, B). Our analysis revealed notable distribution patterns of the *POLRMT* gene across various cell types, with a particular focus on epithelial cells (cancerous cells) and cancer-associated endothelial cells (Fig. 1A, B). Dot plots generated from the dataset indicate that *POLRMT* is predominantly expressed in epithelial and endothelial cells, with a marked increase in expression observed in tumor samples (Fig. 1C), suggesting a potential role for *POLRMT* in tumorigenesis or tumor progression.

We next isolated a subgroup of epithelial cells from the dataset (Fig. 1D). Subsequent analysis revealed that *POLRMT* is significantly overexpressed in the epithelial cells of CMS4 (consensus molecular subtype 4) CRC subtype (Fig. 1E, F). The CMS4 subtype is known for its mesenchymal characteristics and poor prognosis [37], implicating *POLRMT* as a potential biomarker or therapeutic target in this aggressive cancer subtype. We next conducted a detailed analysis of stromal cell populations (Fig. 1G). Within this subgroup, *POLRMT* expression was found to be significantly elevated in cancer-related proliferative endothelial cell subtypes (Fig. 1H, I). Furthermore, POLRMT was also distributed in tip-like and stem-like stromal cells (Fig. 1H, I). These scRNA-seq results confirmed *POLRMT* overexpression in CRC cells.

### POLRMT overexpression in CRC tissues and cells

**Fig. 2:**
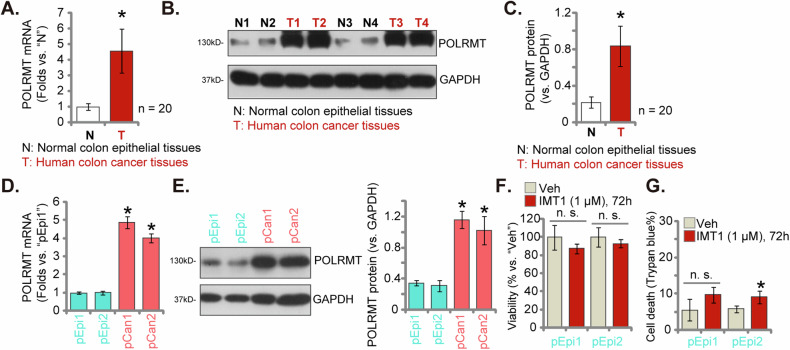
POLRMT overexpression in CRC tissues and cells. *POLRMT* mRNA and protein levels were evaluated in colon cancer tissues (“T”) and corresponding adjacent normal colon tissues (“N”) obtained from a cohort of twenty primary colon cancer patients (**A**–**C**). The expression of *POLRMT* mRNA and protein was assessed in listed primary human colon cancer cells (“pCan1”/”pCan2”) and primary human colon epithelial cells (“pEpi1”/”pEpi2”) (**D**, **E**). “pEpi1”/”pEpi2” cells were subjected to treatment with IMT1 (1 μM) for 72 h, followed by assessments of cell viability using CCK-8 (**F**) and determination of cell death through Trypan blue staining (**G**). The data were presented as mean ± standard deviation (SD, *n* = 5), with *indicating *P* < 0.05 compared to “N” tissues (**A**, **C**) or “pEpi1” (**D**, **E**). *Indicating *P* < 0.05 compared to “Veh” treatment (**G**) and “n. s.” non-statistical difference (*P* > 0.05) (**F**, **G**). The experiments were repeated five times, yielding consistent results.

We investigated the expression of POLRMT in human CRC tissues. Twenty pairs of colon cancer tissues (“T”) along with adjacent normal colon tissues (“N”) were collected from the primary cancer patients who provided written consent at our institutions. These fresh tissues were processed in tissue lysis buffer. To assess mRNA expression, qRT-PCR assays were conducted, revealing that *POLRMT* mRNA levels in colon cancer tissues were over four times higher than those in normal colon tissues (Fig. 2A). Furthermore, analysis of colon cancer tissues from four representative patients (Patient-1# to Patient-4#) demonstrated an upregulation of POLRMT protein (Fig. 2B). Quantitative analysis encompassing data from all twenty patients confirmed a significant elevation of POLRMT protein in colon cancer tissues compared to normal tissues (*P* < 0.05 versus “N” tissues, Fig. 2C).

Additionally, we conducted a comparison of POLRMT expression between CRC cells and normal colon epithelial cells. Both *POLRMT* mRNA and protein levels were notably higher in the primary colon cancer cells (“pCan1” and “pCan2”) in comparison to levels observed in primary human colon epithelial cells (“pEpi1” and “pEpi2”, Fig. 2D, E). These findings collectively affirm the heightened expression of POLRMT in both CRC tissues and cells. We also explored the activity of IMT1 in “pEpi1” and “pEpi2” epithelial cells where POLRMT expression is low. As shown, IMT1 (1 μM, 72 h) only induced minimum viability reduction (Fig. 2F) and cell death (Fig. 2G) in the epithelial cells.

### IMT1 demonstrates substantial anti-CRC cell activity

**Fig. 3:**
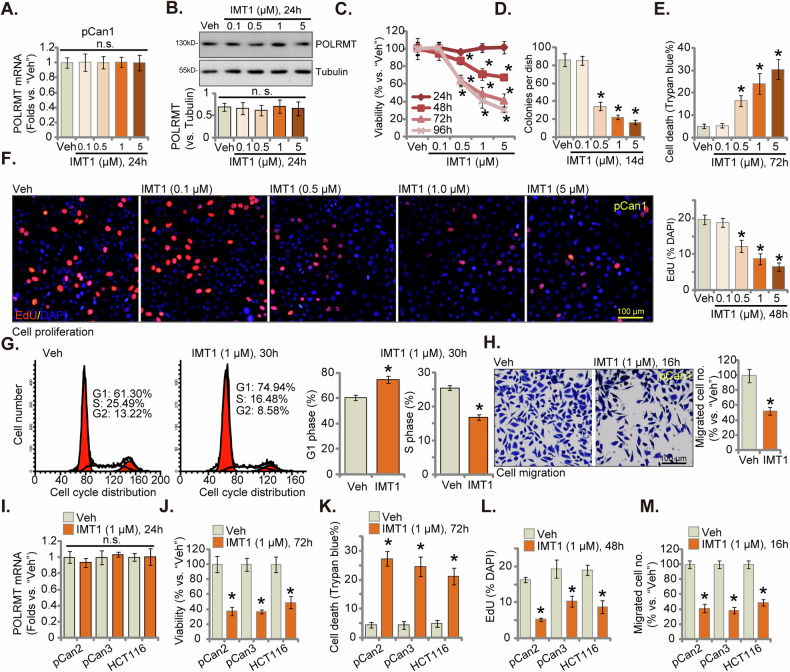
IMT1 demonstrates substantial anti-CRC cell activity. The primary human colon cancer cells, pCan1, underwent treatment with IMT1 at the specified concentrations. Cells were then cultivated for the designated duration, and the assessment included the examination of *POLRMT* mRNA (**A**) and protein (**B**) expression. Additionally, various parameters including cell viability (**C**), colony formation (**D**), cell death (measured by the ratio of Trypan blue-positive cells, **E**), and proliferation (evaluated through nuclear EdU incorporation, **F**) were tested. Cell cycle progression (PI-FACS assays, **G**) and in vitro cell migration (**H**) were evaluated using corresponding assays. Other primary colon cancer cells (“pCan-2/pCan-3”) or immortalized colon cancer cells (HCT116) were exposed to IMT1 (1 μM) for designated duration and assessments included *POLRMT* mRNA expression (**I**), cell viability (**J**), cell death (**K**), proliferation (**L**), and in vitro cell migration (**M**) were carried out using the similar protocols, with results quantified. “Veh” represents the vehicle control (0.1% DMSO). The data were presented as mean ± standard deviation (SD, *n* = 5), with *indicating *P* < 0.05 compared to “Veh” treatment (**B**–**H**, **J**–**M**) and “n. s.” denoting non-statistical difference (*P* > 0.05) (**A**, **I**). The experiments were repeated five times, yielding consistent results. The scale bar is set at 100 μm.

Next, the primary human colon cancer cells, pCan1 (as reported previously [30, 31]) were treated with IMT1 at different concentrations, from 0.1 μM to 5 μM. The concentrations were chosen based on the previous publications [22, 23]. As shown, treatment with the POLRMT inhibitor failed to alter mRNA (Fig. 3A) and protein (Fig. 3B) expression of POLRMT in pCan1 cells. IMT1 exhibited a dose-dependent inhibition of cell viability and a decrease in CCK-8 OD within pCan1 cancer cells (Fig. 3C). Specifically, concentrations of IMT1 at 0.5 μM or higher significantly reduced viability in pCan1 cells, while the lowest concentration (0.1 μM) failed to induce a noticeable effect (Fig. 3C). Furthermore, the POLRMT inhibitor demonstrated a time-dependent response in suppressing viability in pCan1 cells, necessitating a minimum duration of 48 h to exert a significant effect (Fig. 3C). The reduction in viability induced by IMT1 persisted for at least 96 h (Fig. 3C).

Further experimental results demonstrated that IMT1 inhibited pCan1 cell colony formation in a dose-dependent manner and was again significant at 0.5–5 μM (Fig. 3D). Moreover, significant cell death was detected in IMT1 (0.5-5 μM)-treated pCan1 cells, evidenced by the increased ratio of Trypan blue staining cells (Fig. 3E). Additionally, the POLRMT inhibitor also dose-dependently inhibited pCan1 cell proliferation and decreased EdU-positive nuclei percentage (Fig. 3F). The above titration experiments showed that 1 μM of IMT1 exerted significant effect in surpassing cell viability (Fig. 3C) and colony formation (Fig. 3D), inducing cell death (Fig. 3E) and suppressing cell proliferation (Fig. 3F). This concentration was therefore selected for the following experiments.

The PI-FACS assays were thereafter employed to test cell cycle progression. As shown, after IMT1 (1 μM) treatment in pCan1 cells, the G1-phase was significantly increased, whereas the S-phase was decreased (Fig. 3G). These results implied that IMT1 hindered cell cycle progression and induced G1-S arrest in pCan1 primary cells (Fig. 3G). In addition, the POLRMT inhibitor slowed the in vitro cell migration (Fig. 3H) of pCan1 primary cells.

The potential effect of IMT1 in other CRC cells was evaluated as well. In primary colon cancer cells derived from other two patients, pCan2 and pCan3 (as reported previously [30, 31]), as well as in immortalized HCT116 cells, IMT1 (1 μM) treatment again failed to alter *POLRMT* mRNA expression (Fig. 3I). IMT1 (1 μM) did inhibit cell viability, as indicated by the decrease in CCK-8 OD (Fig. 3J), and prompted cell death, evident from the increase in Trypan blue staining (Fig. 3K), across these primary and immortalized CRC cells. Furthermore, the POLRMT inhibitor impeded cell proliferation, as demonstrated by reduced nuclear EdU incorporation (Fig. 3L), and inhibited in vitro cell migration (Fig. 3M) in the CRC cells. Collectively, these findings provide evidence supporting the significant anti-CRC cell activity exhibited by the POLRMT inhibitor.

### IMT1 induces apoptosis activation in CRC cells

**Fig. 4:**
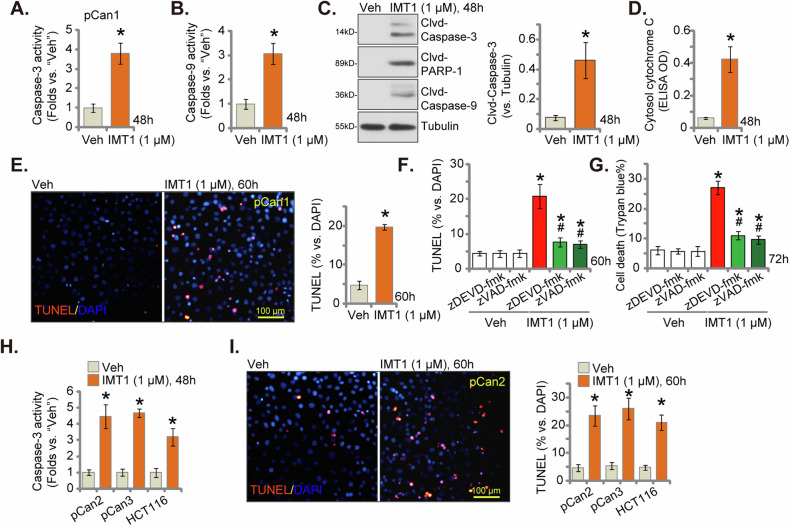
IMT1 induces apoptosis activation in CRC cells. The primary human colon cancer cells, pCan1, underwent treatment with IMT1 (1 μM). Subsequently, they were cultivated for the designated duration, and the assessments including the Caspase-3 activity (**A**) and the Caspase-9 activity (**B**), expression of apoptosis-related proteins (**C**), and cytosol cytochrome C contents (ELISA assays, **D**) were performed. Cell apoptosis was tested through nuclear TUNEL staining assays (**E**). Preceding IMT1 (1 μM) treatment for the specified period, pCan1 cells underwent pretreatment with zDEVD-fmk (50 μM) or zVAD-fmk (50 μM) for 1 h. Subsequent assessments included the evaluation of apoptosis via nuclear TUNEL staining assays (**F**) and the determination of cell death through Trypan blue staining (**G**). Other primary colon cancer cells (“pCan-2/pCan-3”) or immortalized colon cancer cells (HCT116) were exposed to IMT1 (1 μM) for a designated duration and assessments included the Caspase-3 activity (**H**) and nuclear TUNEL staining (**I**). “Veh” represents the vehicle control (0.1% DMSO). The data were presented as mean ± standard deviation (SD, *n* = 5), with *indicating *P* < 0.05 compared to the “Veh” treatment (**A**–**I**) and ^#^indicating *P* < 0.05 compared to the IMT1 only treatment (**F**, **G**). The experiments were repeated five times, yielding consistent results. The scale bar is set at 100 μm.

Considering that IMT1 induced proliferation inhibition and cell cycle arrest in CRC cells, we next tested its effect on cell apoptosis. As shown, in pCan-1 primary cancer cells, IMT1 (1 μM) treatment significantly increased the Caspase-3 activity (Fig. 4A) and the Caspase-9 activity (Fig. 4B). Moreover, the POLRMT inhibitor provoked cleavages of Caspase-3, Caspase-9 and Poly (ADP-ribose) polymerase 1 (PARP1) in pCan1 cells (Fig. 4C). In addition, levels of cytosol cytochrome-C, tested via an ELISA kit, were boosted in IMT1-stimulated pCan1 primary cells (Fig. 4D). These results supported the activation of mitochondrial apoptosis cascade in pCan1 cells [38–40]. Further studies showed that IMT1 (1 μM) induced apoptosis in pCan1 cells and TUNEL-positive nuclei percentage was significantly bolstered (Fig. 4E).

To test the link between apoptosis activation and IMT1-induced cytotoxicity in pCan1 cells, two well-established Caspase-apoptosis inhibitors were employed, including the Caspase-3 specific inhibitor zDEVD-fmk and the pan Caspase inhibitor zVAD-fmk. The TUNEL-nuclei staining assay results showed that co-treatment with the two inhibitors almost blocked IMT1-induced apoptosis activation in pCan-1 cells (Fig. 4F). Importantly, the two significantly mitigated IMT1-caused death (tested via Trypan blue staining assay, Fig. 4G) in pCan1 cells, supporting that apoptosis should be the primary mechanism of IMT1’s cytotoxicity in CRC cells. IMT1 induced similar actions in other primary (pCan2 and pCan3) and immortalized (HCT116) CRC cells. The Caspase-3 activity was strengthened following IMT1 treatment in the CRC cells (Fig. 4H) and apoptosis was activated, which was evidenced by an increased TUNEL-nuclei ratio (Fig. 4I). These results clearly showed that IMT1 provoked apoptosis in CRC cells.

### IMT1 disrupts mitochondrial functions in CRC cells

**Fig. 5:**
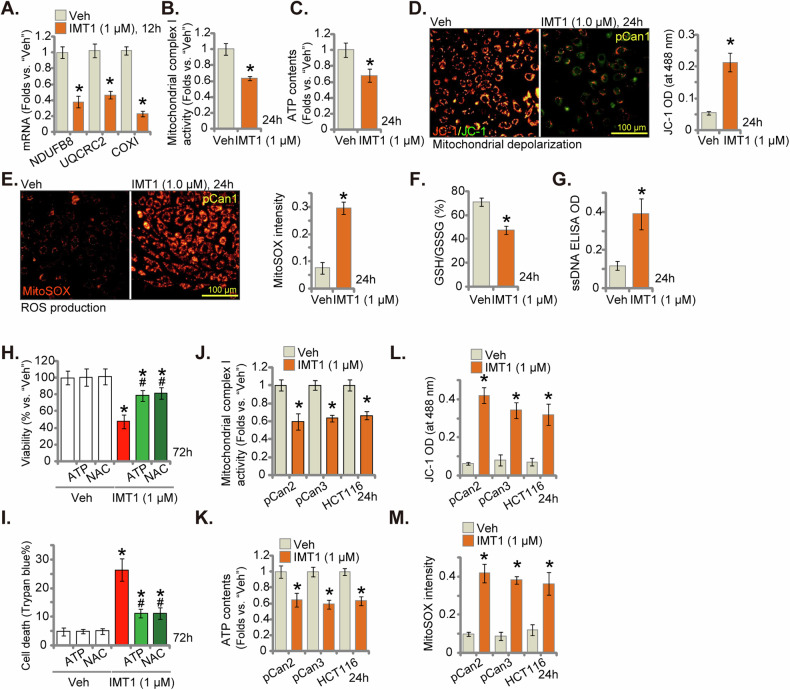
IMT1 disrupts mitochondrial functions in CRC cells. The primary human colon cancer cells, pCan1, underwent treatment with IMT1 (1 μM) for the specified period, and the mRNA expression of *NDUFB8*, *COXI*, and *UQCRC2* was examined using qRT-PCR assays (**A**). Additionally, mitochondrial complex I activity (**B**) and cellular ATP levels (**C**) were tested. Mitochondrial depolarization was assessed through JC-1 fluorescence staining (**D**), while cellular ROS levels were determined using MitoSOX staining (**E**). Furthermore, GSH/GSSH contents were analyzed (**F**), and DNA damage was quantified through ssDNA ELISA (**G**). Preceding IMT1 (1 μM) treatment for the specified period, pCan1 cells underwent pretreatment with NAC (500 μM) or ATP (1 mM) for 0.5 h. Subsequent assessments included the examination of cell viability using CCK-8 (**H**) and the determination of cell death through Trypan blue staining (**I**). Other primary colon cancer cells (“pCan-2/pCan-3”) or immortalized colon cancer cells (HCT116) were exposed to IMT1 (1 μM) for designated duration, mitochondrial complex I activity (**J**), and cellular ATP levels (**K**) were quantified. Mitochondrial depolarization was assessed through JC-1 fluorescence staining (**L**), and cellular ROS levels were determined using MitoSOX fluorescence staining assay (**M**). “Veh” represents the vehicle control (0.1% DMSO). The data were presented as mean ± standard deviation (SD, *n* = 5), with *indicating *P* < 0.05 compared to “Veh” treatment (**A**–**M**) and ^#^indicating *P* < 0.05 compared to the IMT1 only treatment (**H**, **I**). The experiments were repeated five times, yielding consistent results. The scale bar is set at 100 μm.

Next, the potential role of IMT1 on the mitochondrial functions in CRC cells was explored. First, we showed that POLRMT-dependent mitochondrial transcripts, including *NDUFB8*, *UQCRC2* and *COXI* [17–19, 22, 23], were downregulated in IMT1 (1 μM)-treated pCan1 cells (Fig. 5A). Treatment with the POLRMT inhibitor led to downregulation of mitochondrial complex I activity in the primary cancer cells (Fig. 5B), which was accompanied with ATP reduction (Fig. 5C). Mitochondrial depolarization was observed in IMT1-stimulated pCan1 cells, which was evidenced by the transition of JC-1 red aggregates to green monomers (Fig. 5D). With mitochondrial dysfunction, ROS production and oxidative injury were detected in IMT1-treated pCan1 cells, evidenced by increased MitoSOX red fluorescence staining (Fig. 5E). Moreover, GSH/GSSG ratio was decreased in IMT1-treated pCan1 cells (Fig. 5F), further supporting oxidative injury by the POLRMT inhibitor (Fig. 5F). Oxidative stress can induce lesions and modifications in DNA, leading to the formation of ssDNA and breaks. As demonstrated, ssDNA contents were significantly increased in IMT1-stimulated pCan1 cells (Fig. 5G), further providing evidence of oxidative injury by IMT1. Crucially, IMT1-caused cytotoxicity in pCan1 cells can be ameliorated by exogenously added ATP and the antioxidant NAC. NAC or ATP potently inhibited IMT1-induced viability reduction (Fig. 5H) and cell death (Fig. 5I).

Treatment with the POLRMT inhibitor also disrupted mitochondrial functions in other primary colon cancer cells (pCan2 and pCan3) and immortalized HCT116 cells. IMT1 downregulated mitochondrial complex I activity (Fig. 5J) and decreased cellular ATP contents (Fig. 5K) in these primary and immortalized CRC cells. Moreover, mitochondrial depolarization was detected in IMT1-stimulated CRC cells, evidenced by the accumulation of JC-1 green monomers (Fig. 5L). In addition, the increased MitoSOX intensity supported ROS production and oxidative injury in the CRC cells after IMT1 treatment (Fig. 5M). These results together supported that IMT1 disrupted mitochondrial functions in CRC cells.

### POLRMT silencing exerts remarkable anti-cancer cell activity in CRC cells

**Fig. 6:**
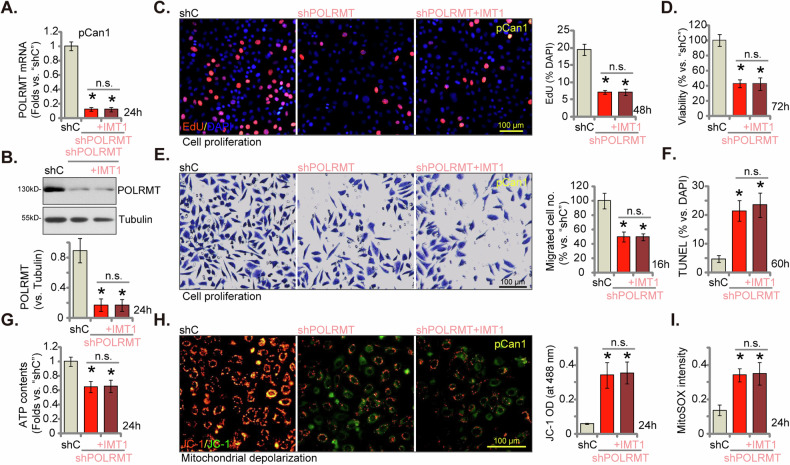
POLRMT silencing exerts remarkable anti-cancer cell activity in CRC cells. The primary colon cancer cells, pCan1, expressing the lentiviral POLRMT shRNA (“shPOLRMT”), were treated with IMT1 (1 μM) and further cultivated for designated time, the expression of *POLRMT* mRNA and protein was assessed (**A**, **B**); Cell proliferation, viability, migration and apoptosis were measured by nuclear EdU staining (**C**), CCK-8 (**D**), “Transwell” (**E**) and nuclear TUNEL staining (**F**) assays, respectively; The cellular ATP contents were quantified as well (**G**); mitochondrial depolarization was assessed through JC-1 fluorescence staining (**H**), with cellular ROS levels determined using MitoSOX fluorescence staining assay (**I**). “shC” stands for cells with scramble control shRNA. The data were presented as mean ± standard deviation (SD, *n* = 5), with *indicating *P* < 0.05 compared to “shC” treatment (**A**–**I**) and “n. s.” denoting non-statistical difference (*P* > 0.05) (**A**-**I**). The experiments were repeated five times, yielding consistent results. The scale bar is set at 100 μm.

Since POLRMT inhibition by IMT1 induced a robust inhibitory effect in CRC cells, we hypothesized that genetic suppression of POLRMT could yield comparable effects. To test this hypothesis, we employed the shRNA technique. Specifically, lentivirus-mediated delivery of POLRMT shRNA was introduced into pCan1 cells, resulting in the establishment of stable cells termed “shPOLRMT” following selection using puromycin. In comparison to pCan1 cells expressing a scrambled control shRNA (“shC”), the shPOLRMT pCan1 cells exhibited reduced expression of both *POLRMT* mRNA (Fig. 6A) and protein (Fig. 6B). Similar to actions by IMT1, POLRMT shRNA also inhibited proliferation (nuclear EdU incorporation, Fig. 6C), viability (CCK-8 OD, Fig. 6D) and migration (tested via “Transwell” assays, Fig. 6E), and provoked apoptosis (tested via nuclear TUNEL staining, Fig. 6F) in pCan1 cells. Importantly, adding IMT1 failed to alter *POLRMT* mRNA (Fig. 6A) and protein (Fig. 6B) expression in shPOLRMT pCan1 cells. Neither did it further inhibit pCan1 cell proliferation (Fig. 6C), viability (Fig. 6D) and migration (Fig. 6E), nor further increasing apoptosis (Fig. 6F). Further studies showed that POLRMT silencing downregulated cellular ATP contents (Fig. 6G), induced mitochondrial mitochondrial depolarization (JC-1 green monomers accumulation, Fig. 6H) and induced ROS production (MitoSOX intensity increasing, Fig. 6I). Such actions by POLRMT shRNA were again not exacerbated by IMT1 treatment (Fig. 6G-I). These results indicated that IMT1 exerted an inhibitory effect in CRC cells via blocking POLRMT.

### POLRMT overexpression exerts cancer-promoting activity in CRC cells

**Fig. 7:**
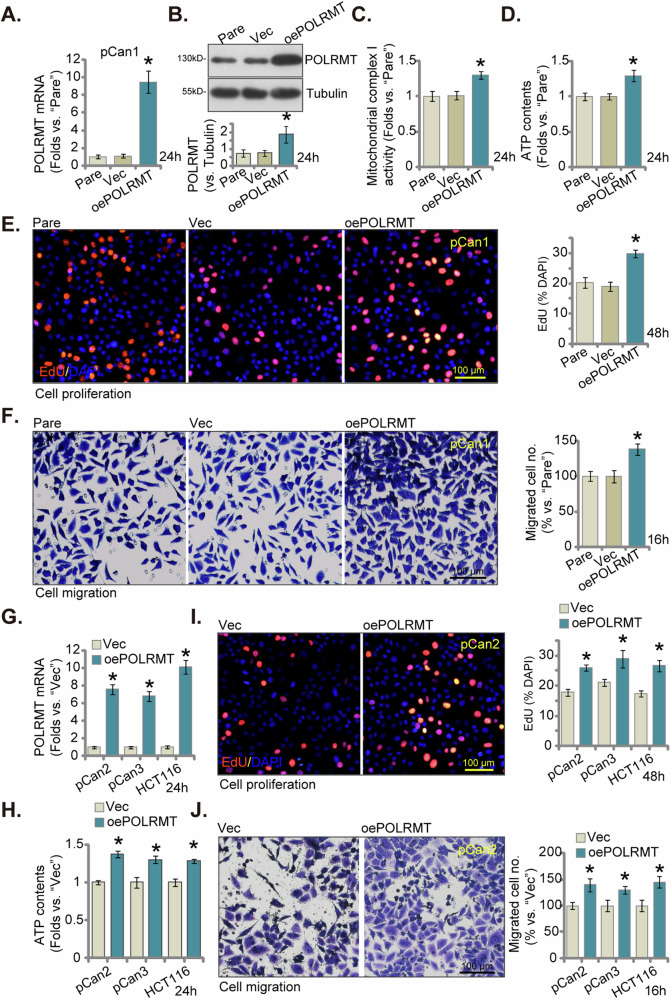
POLRMT overexpression exerts cancer-promoting activity in CRC cells. The primary human colon cancer cells, “pCan-1/-2/-3,” and the immortalized cell line (HCT116) were engineered to express either the lentiviral POLRMT-overexpressing construct (“oePOLRMT”) or the vector (“Vec”). The expression of *POLRMT* mRNA and protein was assessed (**A**, **B**, and **G**). Following cultivation for specified durations, mitochondrial complex I activity (**C**) and cellular ATP levels (**D**, **H**) were quantified. Additionally, measurements were taken for nuclear EdU incorporation (**E**, **I**) and in vitro cell migration (**F**, **J**). “Pare” stands for parental control cells. The data were presented as mean ± standard deviation (SD, *n* = 5), with *indicating *P* < 0.05 compared to “Vec” cells (**A**–**J**). The experiments were repeated five times, yielding consistent results. The scale bar is set at 100 μm.

Building upon the preceding findings, we postulated that the overexpression of POLRMT could potentially exert tumor-promoting effects. To investigate this hypothesis, a lentivirus carrying a construct expressing POLRMT was introduced into pCan1 cells, leading to the establishment of stable cells termed “oePOLRMT” subsequent to selection with puromycin. As compared to control cells with vector (“Vec”), expression of *POLRMT* mRNA (Fig. 7A) and protein (Fig. 7B) was substantially increased in oePOLRMT pCan1 cells. Ectopic overexpression of POLRMT strengthened mitochondrial functions in pCan1 cells by increasing mitochondrial complex I activity (Fig. 7C) and cellular ATP contents (Fig. 7D). Functional assays revealed that the proliferation of pCan1 cells, assessed through EdU-nuclei incorporation, was enhanced subsequent to the overexpression of POLRMT (Fig. 7E). Furthermore, accelerated cell migration (Fig. 7F) was noticed in the oePOLRMT pCan1 cells. The same lentiviral construct was utilized to over-express POLRMT (“oePOLRMT”) in other primary colon cancer cells (pCan2 and pCan3) and immortalized HCT116 cells. *POLRMT* mRNA levels were significantly increased in these oePOLRMT CRC cells (Fig. 7G), where ATP contents were augmented (Fig. 7H). POLRMT overexpression further promoted cell proliferation (nuclear EdU incorporation, Fig. 7I) and accelerated in vitro cell migration (Fig. 7J) in the CRC cells.

### POLRMT inhibition by IMT1 suppresses Akt-mTOR activation in CRC cells

**Fig. 8:**
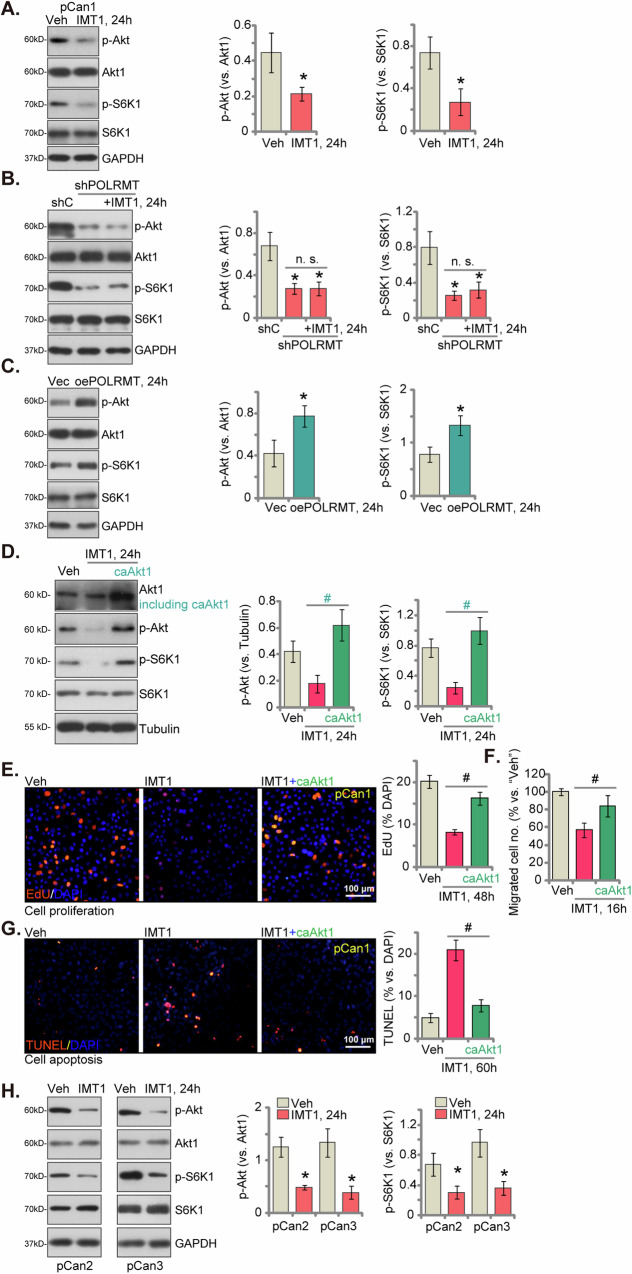
POLRMT inhibition by IMT1 suppresses Akt-mTOR activation in CRC cells. Primary colon cancer cells (pCan1) were subjected to a 24 h treatment with IMT1 (1 μM) or vehicle control (“Veh”), and the protein composition in total cell lysates was analyzed (**A**). pCan1 cells expressing lentiviral POLRMT shRNA (“shPOLRMT”) were exposed to IMT1 (1 μM) and further cultured for 24 h. Additionally, control cells with scramble control shRNA (“shC”) were cultivated for the same duration. The listed protein in total cell lysates was assessed (**B**). pCan1 cells were modified to express either a lentiviral POLRMT-overexpressing construct (“oePOLRMT”) or a vector (“Vec”). These cells were cultured for 24 h, following which the listed protein in total cell lysates was analyzed (**C**). Stable pCan-1 cells containing the constitutively active S473D mutant Akt1 (caAkt1) were treated with IMT1 (1 μM) for specific periods, and control cells were treated with vehicle control (“Veh”). The proteins present in total cell lysates were examined (**D**); cell proliferation, migration, and apoptosis were examined by EdU-nuclei staining (**E**), “Transwell” (**F**), and TUNEL-nuclei staining (**G**) assays, respectively. Other primary colon cancer cells (pCan2 and pCan3) were subjected to a 24 h treatment with IMT1 (1 μM) or vehicle control (“Veh”), and the listed proteins in total cell lysates were analyzed (**H**). The data were presented as mean ± standard deviation (SD, *n* = 5), with *indicating *P* < 0.05 compared to “Veh” (**A**, **H**)/ “shC” (**B**)/“Vec” (**C**) and “n. s.” denoting non-statistical difference (*P* > 0.05) (**B**). ^#^Indicating *P* < 0.05 (**D**-**G**). The experiments were repeated five times, yielding consistent results. The scale bar is set at 100 μm.

The Akt-mTOR pathway is a pivotal signaling cascade implicated in the development and advancement of CRC [41–44]. Increased mitochondrial activity has been identified as a stimulant for Akt-mTOR activation within cancerous cells [45–47]. Consequently, we proceeded to investigate the impact of IMT1 on the Akt-mTOR cascade in CRC cells. Upon treating pCan-1 primary cancer cells with IMT1 (at 1 μM), remarkable suppression of Akt (Ser-473) and ribosomal protein S6 kinase 1 (S6K1, Thr-389) phosphorylation was observed (Fig. 8A), signifying the inhibition of the Akt-mTOR axis. There were no significant alterations in the expression levels of total Akt1 and S6K1 (Fig. 8A). Moreover, the silencing of POLRMT through shRNA (“shPOLRMT”, see Fig. 4) also led to the inhibition of Akt-mTOR pathway activation and a decrease in Akt-S6K1 phosphorylation in pCan-1 cells (Fig. 8B). The expression of Akt1 and S6K1 remained unaltered (Fig. 8B). Notably, the introduction of IMT1 (1 μM, 24 h) into shPOLRMT pCan-1 cells did not result in an additional decrease in Akt-S6K1 phosphorylation (Fig. 8B). Conversely, overexpression of POLRMT led to heightened Akt-mTOR activation in CRC cells. In the pCan-1 cells overexpressing POLRMT (“oePOLRMT”, see Fig. 5), there was an increase in Akt-S6K1 phosphorylation (Fig. 8C), while the levels of total Akt1 and S6K1 remained unaltered (Fig. 8C).

To investigate the impact of Akt-mTOR inactivation on IMT1-induced anti-cancer activity, we introduced the lentivirus carrying a constitutively-active S473D mutant Akt1 (caAkt1) into pCan-1 cells (Fig. 8D). This led to the restoration of Akt-S6K1 phosphorylation in cells treated with IMT1 (Fig. 8D). Functionally, the proliferation arrest induced by IMT1 (shown by reduced EdU-labeled nuclei, Fig. 8E), inhibition of migration (Fig. 8F), and the increase in cell apoptosis (evident by higher TUNEL-labeled nuclei, Fig. 8G) were largely mitigated by the presence of caAkt1. These findings suggest that the suppression of Akt-mTOR signaling plays a crucial role in the anti-CRC cell activity induced by IMT1. In other primary colon cancer cells (pCan2 and pCan3), the administration of IMT1 (1 μM, 24 h) similarly suppressed the phosphorylation of Akt-S6K1 (Fig. 8H), while demonstrating no impact on the overall expression of total Akt-S6K1 (Fig. 8H).

### Oral administrant of IMT1 impedes primary colon cancer xenograft growth in nude mice

**Fig. 9:**
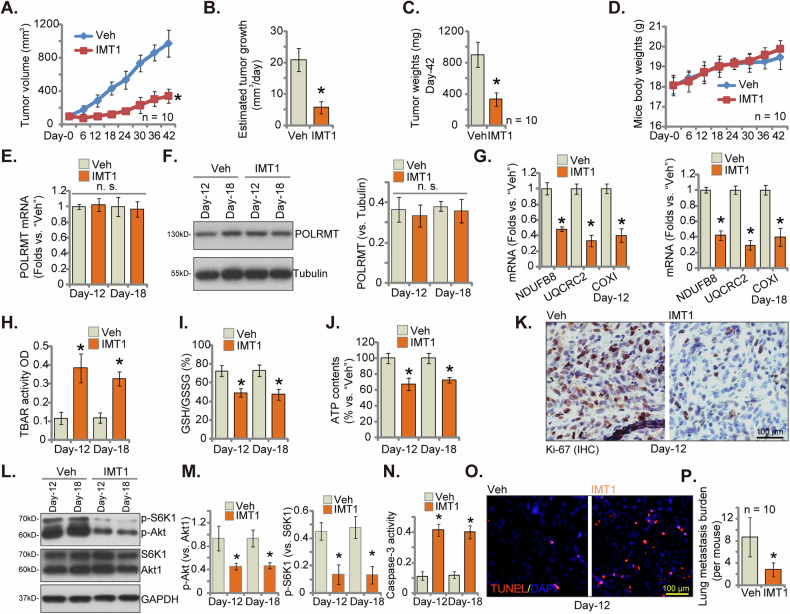
Oral administration of IMT1 impedes primary colon cancer xenograft growth in nude mice. Nude mice bearing pCan1 xenografts were subjected to oral administration of either IMT1 (50 mg/kg body weight) or vehicle control (“Veh”). The volumes of pCan1 xenografts (**A**) and the body weights of the animals (**D**) were recorded at six-day intervals. The daily tumor growth rate, calculated in mm^3^ per day, was determined (**B**). At “Day-42,” all xenograft tumors were isolated and weighed (**C**). Additionally, one pCan1 xenograft from each group was isolated on Day 12 and Day 18 for the analysis of listed mRNA and protein expression (**E**–**G**, **L**, and **M**). TBAR intensity (**H**), GSH/GSSG ratio (**I**), ATP levels (**J**), and Caspase-3 activity (**N**) were also measured. Furthermore, sections of pCan1 xenografts underwent IHC to test nuclear Ki-67 (**K**), as well as immunofluorescence detection of TUNEL-positive nuclei (**O**). Nude mice were injected via tail vein with pCan1 primary cells to establish the lung metastasis model. Mice were then subjected to oral administration of either IMT1 (50 mg/kg body weight) for two cycles (on Day 0 and Day 3) or vehicle control (“Veh”). After 40 days (“Day-40”), the number of lung metastases was quantified (**P**). Data were mean ± standard deviation (SD). In panels **A**–**D** and **P**, every experimental group comprised ten mice (*n* = 10). For panels **E**–**O**, measurements were conducted on five randomly selected tissue pieces within each xenograft (*n* = 5). *Indicating *P* < 0.05 compared to “Veh” treatment (**A**-**E**, **G**–**P**) and “n. s.” denoting non-statistical difference (*P* > 0.05) (**F**). The scale bar is set at 100 μm.

In vivo testing was conducted to evaluate the anti-tumor efficacy of IMT1. Subcutaneous injection of seven million pCan1 primary cells per mouse was performed, leading to the formation of pCan1 xenografts after three weeks, each reaching close to 100 mm^3^, denoted as “Day-0”. Nude mice bearing pCan1 xenografts were then subjected to oral administration of IMT1 at 50 mg/kg body weight or vehicle control, for two cycles (on Day-0 and Day-3). The tumor growth curve demonstrated significant inhibition of pCan1 xenograft growth in mice treated with IMT1 (Fig. 9A). The daily growth rate of pCan1 xenografts, measured in mm [3] per day, markedly decreased with IMT1 administration (Fig. 9B). By Day-42, IMT1-treated pCan1 xenografts were notably smaller and lighter than those treated with the vehicle control (Fig. 9C), further indicating a substantial inhibition of pCan1 xenograft growth in nude mice following IMT1 administration. There were no notable distinctions in the mice’s body weights between the groups treated with IMT1 and those treated with the vehicle (Fig. 9D). No apparent toxicities were observed in IMT1-treated mice, consistent with previous findings [21–23, 35].

On Day 12 and Day 18, one pCan1 xenograft from each group was isolated, and xenograft tissues were tested. Analysis of signaling changes in xenograft tissues revealed that IMT1 did not alter *POLRMT* mRNA (Fig. 9E) and protein (Fig. 9F) expression in pCan1 xenograft tissues. Yet, there was a marked decrease in mitochondrial transcripts dependent on POLRMT, such as *NDUFB8*, *UQCRC2*, and *COXI*, in the pCan1 xenograft tissues treated with IMT1 (Fig. 9G). Compared to vehicle-treated control xenografts, the TBAR intensity (Fig. 9H) was significantly higher in IMT1-treated pCan1 xenograft, where the GSH/GSSG ratio was decreased (Fig. 9I). Additionally, ATP contents were decreased in IMT1-administered xenograft tissues (Fig. 9J). These results supported impaired mitochondrial functions in pCan1 xenografts in response to IMT1 administration.

Additional IHC staining in xenograft sections revealed a decrease in nuclear Ki67 staining within the pCan1 xenograft tissues following IMT1 treatment, indicating a decreased proliferation in vivo (Fig. 9K). Moreover, the Western blotting assay exhibited significant reductions in p-Akt and p-S6K1 levels in the IMT1-treated xenograft tissues (Fig. 9L, M). Supporting apoptosis induction, we showed that the Caspase-3 activity was elevated in IMT1-administered tumor tissues (Fig. 9N). Moreover, tissue immunofluorescence staining demonstrated a substantial increase in nuclear TUNEL staining in the IMT1-treated xenograft slides (Fig. 9O). These results collectively illustrate that administration of IMT1 results in mitochondrial dysfunction, Akt-mTOR pathway inactivation, proliferation inhibition, and apoptosis induction within pCan1 xenografts. At last, a lung metastasis model was developed by injecting pCan1 primary cells into the tail veins of nude mice. As demonstrated in Fig. 9P, oral administration with IMT1 resulted in a significant reduction in lung metastatic burden compared to the control group, indicating IMT1’s potency in inhibiting the metastasis of CRC cells.

## Discussion

Increasing attention is being directed towards understanding the role of mitochondria in cancer, driven by their involvement in cellular metabolism, apoptotic regulation, maintenance of redox balance, and activation of the integrated stress response and immune regulation [6, 7, 48–50]. Mitochondria possess their own DNA (mtDNA), which requires transcription and translation to produce RNAs and proteins essential for mitochondrial function [51–53]. mtDNA-encoded genes are vital for mitochondrial translation, OXPHOS, and other functions [51–53]. Various pharmacological agents have been developed to target these mitochondrial processes, including inhibitors of the electron transport chain (e.g., metformin, rotenone, and oligomycin), tricarboxylic acid (TCA) cycle inhibitors (e.g., CPI-613 and AGI-519), and inhibitors of mitochondrial translation (e.g., doxycycline and tetracycline) [51–55]. Some of these compounds exhibit clinical potential for treating cancer [6, 7, 48–50, 52, 56–58].

Recent findings supported that POLRMT may serve as a pivotal oncogenic gene across various cancer types. Its heightened expression plays a crucial role in facilitating OXPHOS, mitochondrial biogenesis, and the proliferation of diverse cancer cells. Zhou et al., demonstrated a significant elevation of POLRMT expression in both tissues and cells of NSCLC, emphasizing its necessity for NSCLC cell growth [17]. Conversely, the disruption of POLRMT through silencing or KO adversely affected mitochondrial functions, leading to impaired ATP production and subsequent suppression of NSCLC proliferation, migration, and invasion [17]. Han et al., revealed an upregulation of POLRMT mRNA and protein expression in prostate cancer tissues and cells [18]. Depletion of POLRMT hindered OS cell proliferation and migration in vitro, while also inhibiting OS xenograft growth in nude mice [18]. In addition, Wang et al., reported POLRMT overexpression in skin squamous cell carcinoma (SCC) tissues and cells, underscoring its essential role in mitochondrial function and the growth of skin SCC cells [23]. Moreover, Li et al., uncovered an upregulation of POLRMT in prostate cancer, highlighting its significance for cancer cell growth in vitro and xenograft growth in nude mice [35].

**Fig. 10:**
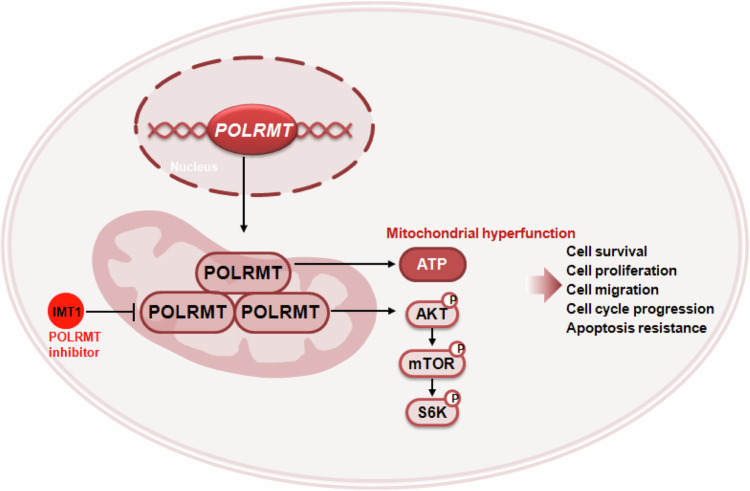
The signaling carton of the study. IMT1 blocks POLRMT activity, disrupts mitochondrial function, and hinders Akt-mTOR activation, thereby strongly inhibiting CRC cell growth in vitro and in vivo.

Here the scRNA seq data revealed that *POLRMT* is overexpressed in CRC cells. Additionally, elevated POLRMT expression was observed in local CRC tissues and cells, revealing the effectiveness of IMT1 in strongly suppressing CRC cell growth. In primary and immortalized CRC cells, IMT1 exhibited profound inhibitory effects on various cell behaviors, including colony formation, cell viability, proliferation, cell cycle progression, and migration. Additionally, IMT1 prompted apoptosis and disrupted mitochondrial function in CRC cells, leading to mitochondrial depolarization, oxidative damage, and reduced ATP levels. In vivo studies showed that oral administration of IMT1 in nude mice effectively hindered primary colon cancer xenograft growth. In addition, POLRMT inactivation, mitochondrial dysfunction, proliferation inhibition, and apoptosis were detected in IMT1-treated xenograft tissues. IMT1 administration also suppressed lung metastasis of primary colon cancer cells in nude mice. Importantly, silencing POLRMT using targeted shRNA mirrored IMT1’s effects, demonstrating robust anti-CRC cell activity. IMT1’s efficacy diminished when POLRMT was silenced in CRC cells, underscoring its dependence on POLRMT. Conversely, augmenting POLRMT expression amplified CRC cell proliferation and migration. These findings suggest a promising therapeutic strategy for CRC by targeting POLRMT with IMT1 (see proposed signaling carton in Fig. 10).

A few advantages of this compound compared to other targeted agents are noteworthy. Firstly, it targets POLRMT, a novel therapeutic oncotarget that is significantly overexpressed in CRC, offering a unique mechanism of action that directly addresses a critical aspect of CRC pathology. Secondly, IMT1 is a first-in-class and potent allosteric inhibitor of POLRMT, demonstrating remarkable specificity, which possibly minimizes off-target effects and enhances therapeutic efficacy [21]. Thirdly, our study here and others [21, 22] have shown that IMT1 exhibits no significant cytotoxicity when administered to mice, highlighting its favorable safety profile and potential for high tolerability in clinical settings.

Mitochondrial hyperfunction has been identified as a potential driver of Akt-mTOR activation. Mitochondrial hyper-function, characterized by elevated ATP production and biosynthetic capacity, can activate Akt signaling in cancer cells [45–47, 59–62]. Enhanced ATP levels provide the necessary energy for Akt activation via phosphorylation [63, 64]. Concomitantly, mitochondrial hyperfunction leads to increased production of metabolic intermediates, including citrate and succinate, which can positively influence Akt-mTOR signaling [65–67]. Enhanced mitochondrial function supports the augmented synthesis of proteins and lipids essential for cellular growth and proliferation. This metabolic environment primes cells for heightened responsiveness to growth factors, thereby enhancing PI3K/Akt pathway activation [68, 69]. Recent studies have highlighted the significance of specific mitochondrial proteins, such as ADCK2 (aarF domain-containing kinase 2) [47], YME1L (YME1 Like 1) [45], TIMM13 (translocase of inner mitochondrial membrane 13) [62] in maintaining mitochondrial functions and their role in activating Akt-mTOR pathways within cancerous cells.

Therefore, one key finding of the study is that IMT1 treatment or POLRMT silencing in primary CRC cells significantly decreased the phosphorylation of Akt1-S6K, whereas POLRMT overexpression had the opposite effect. Akt-mTOR inactivation was also detected in IMT1-treated pCan1 xenografts. Reinstating Akt-mTOR activation by caAkt1 alleviated the cytotoxic effects induced by IMT1 in CRC cells. Thus, IMT1 not only disrupted mitochondrial function, but also hindered Akt-mTOR activation in CRC cells. This implies a potential interplay or cross-regulation between mitochondrial function and Akt signaling pathways, suggesting that targeting POLRMT with IMT1 might influence both mitochondrial activity and downstream Akt-mTOR signaling pathways, thereby strongly inhibiting CRC cell growth in vitro and in vivo (Fig. 10).

## Supplementary information

Figure S1.

## Data Availability

All data are available upon reasonable request.
