# Supplementary material for: Targeting POLRMT by IMT1 inhibits colorectal cancer cell growth
Source: Cell Death Dis. 2024 Sep 3;15(9):643. doi: 10.1038/s41419-024-07023-8 (PMC11372113; doi:10.1038/s41419-024-07023-8)
Supplement: Supplementary file 1 — Figure S1. [file 41419_2024_7023_MOESM1_ESM.pdf]

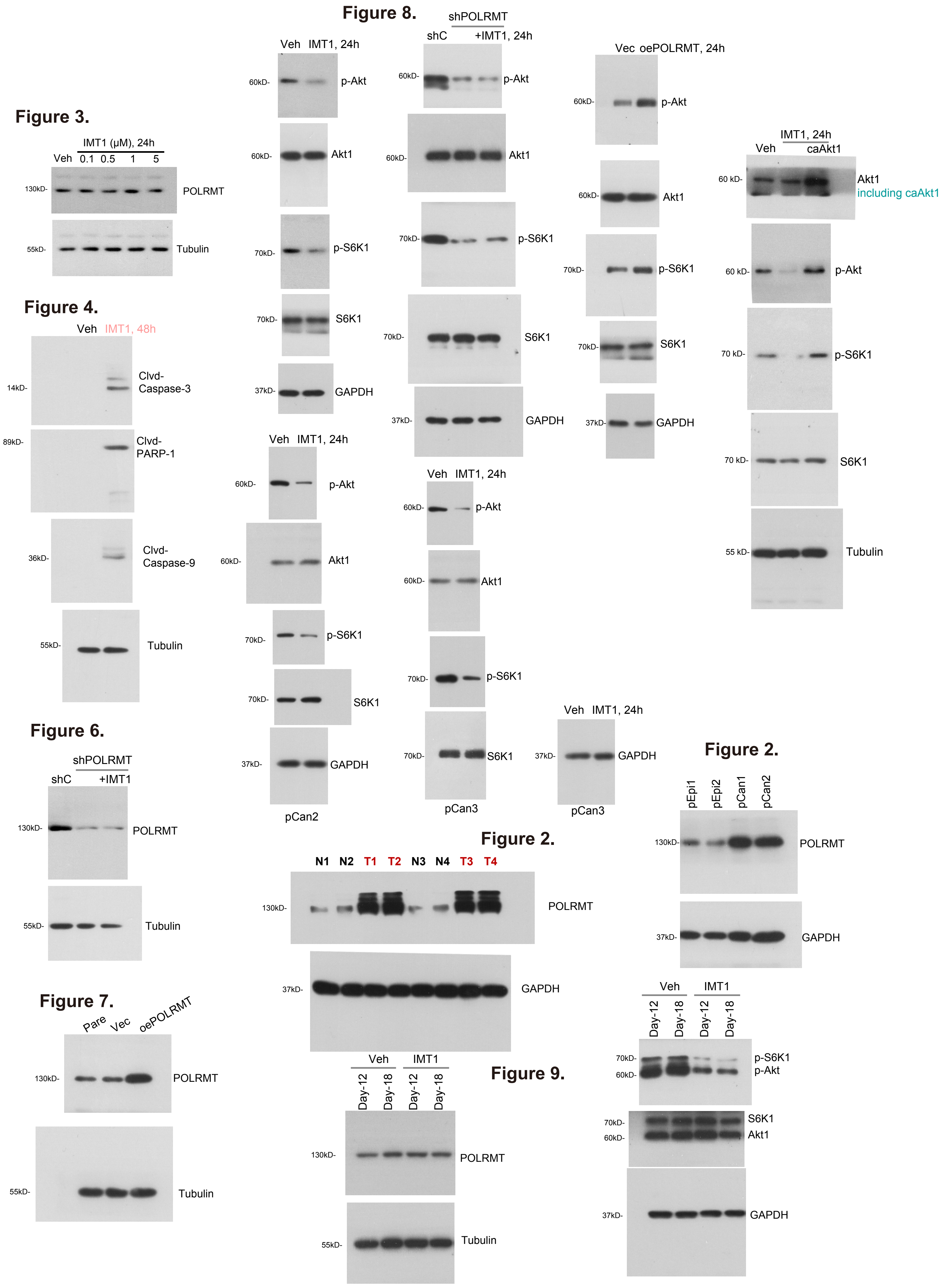

Figure S1. The uncropped blotting images of the study.
The uncropped blotting images corresponding to the cropped data presented in the listed Figures of the study.
